# Robotic versus laparoscopic Roux-en-Y gastric bypass: a propensity-matched comparison of the inflammatory response and MASLD trajectories

**DOI:** 10.1007/s11701-026-03462-x

**Published:** 2026-05-26

**Authors:** Christopher Tuffs, Moschos Delistamatis, Annette Hauenschild, Lisa Sauerbier, Daniar Amin, Alen Kosovic, Anca-Laura Amati, Jens Uwe Albrecht, Ingolf Askevold, Thilo Sprenger, Martin Schneider, Moritz J. Strowitzki

**Affiliations:** 1 Justus-Liebig-University Giessen, Giessen, Germany; 2 Department of General and Visceral Surgery, Hochwald Hospital, Bad Nauheim, Germany; 3 Department of Bariatric and Metabolic Surgery, Knappschaft Clinics Recklinghausen, Recklinghausen, Germany; 4 Department of General and Visceral Surgery, Asklepios Clinic Lich, Lich, Germany; 5 Department of General, Visceral, Thoracic, and Transplantation Surgery, University Hospital Giessen, Giessen, Germany

**Keywords:** Metabolic bariatric surgery, Robotic surgery, Roux-en-Y gastric bypass, Postoperative inflammation, Postoperative systemic inflammatory response, Metabolic dysfunction-associated steatotic liver disease (MASLD)

## Abstract

Roux-en-Y gastric bypass (RYGB) is among the most common surgical procedures for treatment of obesity and metabolic comorbidities and routinely performed using either a laparoscopic- (L-RYGB) or a robotic (R-RYGB) approach. Although overall outcomes are comparable, robotic surgery has been proposed to attenuate postoperative inflammation, which may influence metabolic outcomes. The impact of robotic surgery in RYGB and potential implications for metabolic dysfunction-associated steatotic liver disease (MASLD) remain unclear. We conducted a retrospective cohort study of 306 patients undergoing L- or R-RYGB. Propensity score matching (PSM) was performed to create balanced cohorts with comparable baseline characteristics. We evaluated the postoperative inflammatory response using leukocyte counts and C-reactive protein (CRP), and assessed short-term clinical outcomes, including pain and length of stay (LOS). MASLD trajectories using non-invasive fibrosis scores (NITs) and weight loss were analyzed over one year. After  (PSM), R-RYGB was not associated with an altered inflammatory response with comparable postoperative leukocyte and CRP profiles between both approaches. Patients undergoing R-RYGB reported lower postoperative pain and had a shorter LOS, whereas length of surgery was shorter in the L-RYGB group. Weight loss outcomes and MASLD trajectories assessed by NITs were similar one year post-surgery. L-RYGB and R-RYGB demonstrate comparable postoperative inflammatory responses with similar MASLD trajectories. R-RYGB may offer advantages in the early postoperative recovery at the expense of longer length of surgery.

## Introduction

Metabolic bariatric surgery (MBS) has emerged as the most effective treatment for severe obesity and obesity-associated comorbidities. Comorbidities include arterial hypertension, obstructive sleep apnea, type 2 diabetes mellitus (T2DM) and metabolic dysfunction-associated steatotic liver disease (MASLD), all of which go into remission through MBS [1–3]. Roux-en-Y gastric bypass (RYGB) is among the predominantly performed surgical approaches in MBS, offering excellent weight loss, treatment of comorbidities, and low perioperative risk [4, 5] and is performed as minimally-invasive procedures, either using a laparoscopic (L-RYGB) or robotic approach (R-RYGB). In a meta-analysis investigating short- and long-term outcome after RYGB, R-RYGB was associated with prolonged length of surgery, while the risk for perioperative morbidity, weight-loss efficacy, and remission of comorbidities were unaffected when compared to L-RYGB [6]. However, these mostly retrospective studies did not match patients for baseline characteristics and were thus prone to selection bias.

In contrast, previous studies, investigating the impact of a robotic approach on postoperative inflammation in oncological diseases, describe an attenuated inflammatory response subsequent to surgery compared to a laparoscopic approach [7, 8]. Changes in the postoperative inflammatory response between the robotic and laparoscopic approaches may be attributed to less tissue manipulation utilizing a robotic approach, which reduces the release of proinflammatory cytokines. However, they may also be caused by differences in perioperative care [9]. Attenuation of postoperative inflammation is biologically and clinically relevant, as increased postoperative inflammation is associated with increased blood loss, surgical (and non-surgical) complications and liver injury, as well as poor long-term outcomes [10]. In MBS specifically, postoperative inflammation relates to perioperative morbidity [11], as well as impaired weight loss and glycemic control [12–14]. Thus, postoperative surgery-inflicted inflammation affects the short- and long-term outcome of patients after oncological and bariatric surgery.

MASLD, a disease characterized by chronic hepatic inflammation, improves following bariatric surgery [15, 16]. However, the relationship between the postoperative inflammatory response and MASLD trajectories after MBS has not been systematically studied. Unlike in surgical oncology, in MBS, it remains unclear whether R-RYGB attenuates postoperative inflammation compared to L-RYGB and whether such potential differences impact MASLD trajectories.

We hypothesized that R-RYGB is associated with a reduced postoperative systemic inflammatory response and, consequently, with more favorable MASLD trajectories compared to L-RYGB. We therefore compared R- and L-RYGB with respect to postoperative outcomes and MASLD-related trajectories. Perioperative and short-term outcomes were likewise analyzed to detect differences between L- and R-RYGB.

## Patients and methods

The study was approved by the Ethics Committee of the Justus Liebig University Giessen (Approval number: AZ41/25) and carried out under compliance with the Helsinki Declaration of 1975 (as revised in 1996). Indication and treatment by MBS were performed multidisciplinary and according to the latest national guidelines on obesity as well as metabolic and bariatric surgery.

### Patient selection criteria

All patient data were collected prospectively as part of the metabolic and bariatric surgery program in Giessen (MetaBoGi). We conducted a retrospective cohort analysis of patients undergoing primary RYGB between 2019 and 2025. In total 306 patients were identified during this period.

### Surgical technique

RYGB was performed using either a laparoscopic or robotic minimally invasive approach under general anesthesia with single-shot antibiotic prophylaxis. Patients were positioned supine in the French position. Capnoperitoneum was established and trocar placement was performed accordant to the respective technique. In both approaches, the ligament of Treitz was identified and the jejunum was divided distal to Treitz to create the biliopancreatic limb. The biliopancreatic limb length was determined according to patient-specific and operative considerations, including BMI, weight and height, as well as prevalence and burden of metabolic comorbidities. A 30–40 ml gastric pouch was constructed along the lesser curvature using linear staplers. A gastrojejunostomy was performed either stapled or hand-sewn, followed by measurement of the alimentary limb. A side-to-side jejunostomy was created using a linear staple and mesenteric defects (e.g., Brolin) were routinely closed using running sutures.

### Postoperative care and follow-up protocol

After surgery, patients were routinely monitored in the post-anesthesia care unit and transferred to the surgical ward on the day of surgery. Postoperative management followed a standardized institutional protocol and was applied uniformly in both the robotic and laparoscopic group. This included multimodal analgesia with non-opioid drugs (mostly metamizole and paracetamol) and opioids as required, proton-pump inhibitor therapy (pantoprazole), and weight-adjusted low-molecular weight heparin (mostly enoxaparin sodium) for thromboprophylaxis starting on the day of operation. Patients were typically discharged on postoperative day 2 or 3 once predefined discharge criteria were met, including adequate oral fluid intake, sufficient mobilization, pain control with oral analgesics, and absence of signs of postoperative complications. Before discharge, all patients received structured dietary counseling by certified dieticians. All patients received standardized recommendations for lifelong micronutrient supplementation and nutritional follow-up in accordance with institutional protocols. Scheduled outpatient follow-up visits, including assessment of body weight, metabolic parameters, comorbidities, and medication use, were conducted 3, 6, 9, 12, 18, 24, 30 months postoperatively and annually thereafter. Follow-up adherence at 1 year was 89%.

### Study variables and definitions

Patients were retrospectively stratified for the respective operative technique, resulting in 103 patients who underwent L-RYGB and 203 patients who underwent R-RYGB between 2019 and 2025. Clinical parameters and bloodwork were collected and analysed at preoperative baseline (preoperative), postoperatively, 3 months (3 M), 6 months (6 M) and 1 year (1Y) after surgery. The postoperative systemic inflammatory response was measured by leucocyte count (giga/l) and CRP levels (mg/l). When applicable, visits were assigned to time points using routine clinical windows (e.g., ± 4–8 weeks). Length of stay (LOS) was defined as the interval from hospital admission to discharge measured in days. Body mass index (BMI) was calculated as weight (kg) / height (m^2^). Total weight loss (%TWL) was defined as %TWL = 100 × (weight_preoperative_ (kg) – weight_follow-ups_ (kg)) / (weight_preoperative_ (kg)). Excess body weight loss (%EWL) was defined as %EWL = (weight_preoperative_ (kg) – weight_follow-ups_ (kg)) / (weight_preoperative_ (kg) – ideal weight (kg)) × 100. Ideal weight was defined as: Ideal weight = 25 × (height in m)^2^. T2DM was defined as an HbA1c > 6.5% or current diagnosis of T2DM. Pain while resting or in movement reported by the patient was routinely assessed using a Visual Analogue Scale (VAS) ranging from 0 (no pain) to 10 (worst possible pain).

### Calculation of non-invasive tests for MASH and liver fibrosis

Non-invasive tests (NITs) are routinely used for diagnosis and surveillance of MASH and liver fibrosis [17–21] by combining clinical parameters and blood values into a risk score. They have been validated as a screening tool in the general and obese populations and have been utilized to detect partial or complete remission of MASLD after MBS [22, 23]. NITs were calculated using the following formulas:

$$\begin{aligned}{\boldsymbol{N}}{\boldsymbol{F}}{\boldsymbol{S}} &= -1.675+0.037*age\, \left(years\right)+0.094*BMI\, \left(\frac{kg}{m2}\right) \\& \quad +1.13*diabetes\, \left(yes=1, no=0 \right)+0.99*\frac{AST}{ALT}ratio \\& \quad-0.013*platelet\,count \left(*\frac{{10}^{9}}{L}\right)-0.66*albumin\, \left(\frac{g}{dl}\right) \end{aligned}$$

$${\boldsymbol{F}}{\boldsymbol{I}}{\boldsymbol{B}}-4=\frac{age\, \left(years\right)*AST\, \left(\frac{U}{L}\right)}{platelet\, count \left(\frac{{10}^{9}}{L}\right)* \sqrt{ALT\, \left(\frac{U}{L}\right)}}$$

$${\boldsymbol{A}}{\boldsymbol{P}}{\boldsymbol{R}}{\boldsymbol{I}}=\frac{\frac{AST\, \left(\frac{U}{L}\right)}{40 \left(\frac{U}{L}\right)}}{Platelet\, count \,\left(\frac{{10}^{9}}{L}\right)} * 100$$

$$\begin{aligned}{\boldsymbol{S}}{\boldsymbol{A}}{\boldsymbol{F}}{\boldsymbol{E}} & =2.97*age+5.99*BMI \,\left(if BMI\ge 40\, it\, was\, set\, to\, 40\right)\\& \quad+62.85*diabetes \left(0 \,if\, absent, 1\, if\, present\right) \\& \quad +154.85*Ln\left(AST\right)\\& \quad-58.23*Ln\left(ALT\right)\\& \quad+195. 48*Ln\left(globulin \left(\frac{g}{dl}\right)\right) \\& \quad -141.61* Ln (platelets \left(\frac{{10}^{9}}{\mu l}\right)\\& \quad-75 \end{aligned}$$

$$\begin{aligned}{\boldsymbol{N}}{\boldsymbol{I}}-{\boldsymbol{N}}{\boldsymbol{A}}{\boldsymbol{S}}{\boldsymbol{H}}\,{\boldsymbol{D}}{\boldsymbol{S}}&=-32.771+0.227*BMI \left(\frac{kg}{m2}\right)\\ & \quad+0.062*ALT \left(\frac{U}{L}\right)+0.024*TG\left(\frac{mg}{dl}\right)\\ & \quad+3.881*albumin\left(\frac{g}{dl}\right)\end{aligned}$$

### Statistics

**Fig. 1:**
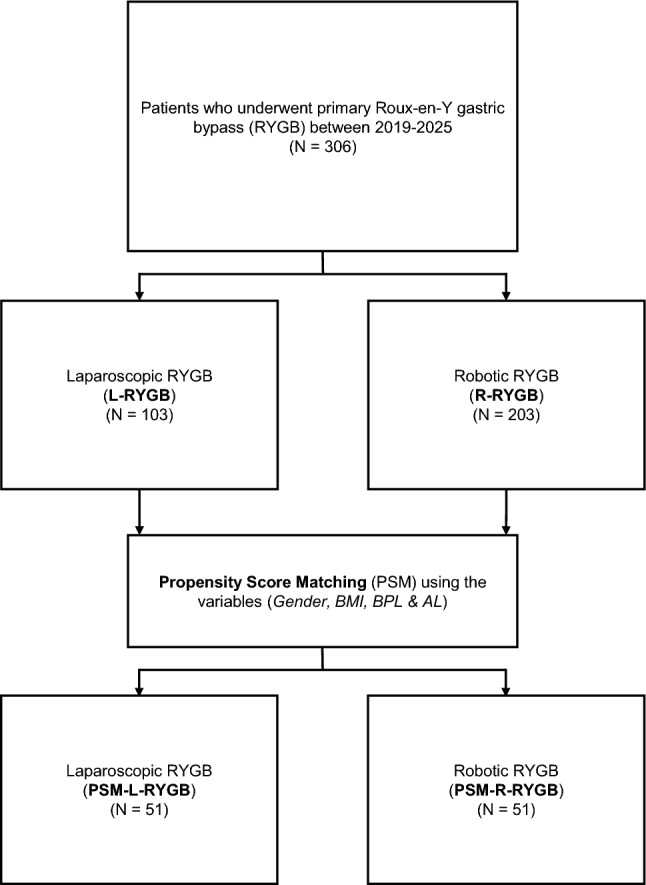
Patient cohort and propensity score matching (PSM) flow diagram. Flow chart of patients who underwent primary Roux-En-Y gastric bypass (RYGB) between 2019–2025 (N = 306). Patients were stratified according to the surgical approach (L-RYGB *versus* R-RYGB). Afterwards, PSM was performed to reduce selection bias and heterogeneity of important baseline patient characteristics, such as gender, preoperative body-mass index (BMI), biliopancreatic limb (BPL) length, and alimentary limb (AL) length, resulting in 1:1 matched groups with an absolute mean difference of < 0.1 (PSM-L-RYGB and PSM-R-RYGB; N = 51 per group)

**Fig. 2:**
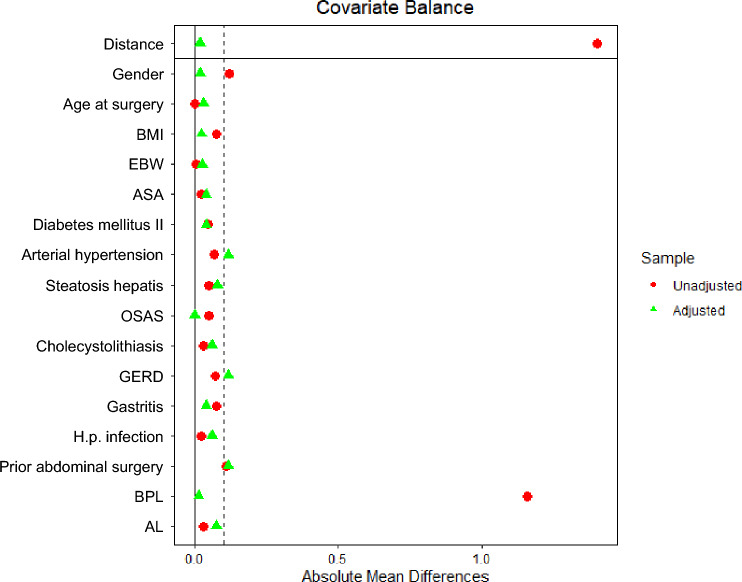
Covariate balance of baseline variables before and after PSM. Love plot displaying absolute standardized mean differences (SMDs) for baseline covariates before (red) and after PSM (green). The dashed vertical line indicated the pre-specified balance threshold (SMD = 0.1)

Continuous variables were depicted as the mean ± standard deviation and categorical variables as number of patients with percentages (rounded to whole numbers). For unmatched between-group comparisons of continuous variables, normality was assessed within each group using the Shapiro-Wilk test (p > 0.05). If both groups were approximately normally distributed, group means were compared using a two-sided Welch t-test. If a group was not normally distributed, the Mann Whitney U test was used. For categorical variables, differences were tested using the Chi-squared test or Fisher´s exact test. To address confounding and selection bias, propensity score matching (PSM) was performed using 1:1 nearest-neighbour matching on the logit propensity score with a caliper of 0.2, as previously described [24, 25], using biliodigestive limb length, alimentary limb length, sex, and preoperative BMI as matching covariates (see Fig. 1). Covariate balance was assessed by calculating the absolute mean difference, visualized in a love plot (see Fig. 2). For the matched cohort, continuous variables were compared on within-pair differences using a paired t-test when differences were approximately normal (Shapiro-Wilk p > 0.05), otherwise using the Wilcoxon signed-rank test. Paired categorical outcomes were compared using McNemar´s test for binary variables; for variables with > 2 categories, a Stuart-Maxwell test was applied. All analyses were conducted in RStudio (Posit PBC, Boston, Massachusetts, USA, Posit Software, Version 2024.09.1 + 394) using readxl, dplyr, tidyr, tibble, string, janitor, gtsummary, MatchIt, cobalt, gt, gtsummary, and rmarkdown. A two-sided p-value < 0.05 was considered statistically significant.

## Results

### Baseline patient characteristics before and after PSM

After stratification of patients who underwent minimally invasive RYGB between 2019 and 2025 for the minimally-invasive approach, 103 patients were allocated to the L-RYGB and 203 patients to the R-RYGB group (see Fig. 1). Before matching, both groups were similar in regard to age, preoperative body-mass index (BMI) and comorbidity profile, but differed in the distribution of gender and length of the biliopancreatic limb, with the L-RYGB group displaying a lower proportion of male patients (17% vs. 29%; p = 0.029), and shorter BPL (71.41 ± 22.58 cm vs. 97.59 ± 14.81 cm; p < 0.001) (see Table 1).

**Table 1:** Baseline patient characteristics before and after propensity score matching (PSM)

Variable	Unmatched cohort			PSM cohort		
	L-RYGB N = 103^1^	R-RYGB N = 203^1^	p-value	PSM-L-RYGB N = 51^1^	PSM-R-RYGB N = 51^1^	p-value
Gender			0.029			> 0.9
Male	17 (17%)	58 (29%)		9 (18%)	10 (20%)	
Female	86 (83%)	145 (71%)		42 (82%)	41 (80%)	
Age (years)	42.82 (11.82)	42.83 (10.86)	> 0.9	45.10 (10.39)	44.73 (11.28)	0.9
BMI	49.08 (6.26)	48.62 (5.70)	0.7	48.74 (7.08)	48.59 (6.31)	> 0.9
EBW	67.30 (19.58)	67.38 (17.19)	> 0.9	66.22 (21.50)	65.70 (18.66)	0.8
ASA score			0.8			0.8
2	49 (48%)	92 (45%)		22 (43%)	20 (39%)	
3	54 (52%)	111 (55%)		29 (57%)	31 (61%)	
Diabetes mellitus II			0.5			0.8
Yes	34 (33%)	76 (37%)		18 (35%)	20 (39%)	
No	69 (67%)	127 (63%)		33 (65%)	31 (61%)	
Arterial hypertension			0.3			0.3
Yes	52 (50%)	116 (57%)		25 (49%)	31 (61%)	
No	51 (50%)	87 (43%)		26 (51%)	20 (39%)	
Steatosis hepatis			0.5			0.6
Yes	31 (30%)	71 (35%)		22 (43%)	18 (35%)	
No	72 (70%)	132 (65%)		29 (57%)	33 (65%)	
OSAS			0.3			0.2
With CPAP	9 (9.0%)	27 (13%)		3 (6.0%)	6 (12%)	
Without CPAP	22 (21%)	31 (15%)		16 (31%)	10 (20%)	
No	72 (70%)	145 (71%)		32 (63%)	35 (69%)	
Cholecystolithiasis			0.5			0.4
Yes	8 (8.0%)	10 (5.0%)		2 (5.0%)	5 (10%)	
No	95 (92%)	193 (95%)		49 (96%)	46 (90%)	
GERD			0.5			0.6
0	83 (81%)	149 (73%)		44 (86%)	38 (75%)	
A	14 (14%)	40 (20%)		6 (12%)	10 (20%)	
B	5 (5.0%)	8 (4.0%)		1 (2.0%)	1 (2.0%)	
C	1 (1.0%)	5 (3.0%)		0 (0%)	1 (2.0%)	
D	0 (0%)	1 (0.5%)		0 (0%)	1 (2.0%)	
Hiatus hernia			0.4			0.8
Yes	18 (17%)	45 (22%)		8 (16%)	10 (20%)	
No	85 (83%)	158 (78%)		43 (84%)	41 (80%)	
Gastritis			0.3			0.8
Yes	64 (62%)	111 (55%)		31 (61%)	29 (57%)	
No	39 (38%)	92 (45%)		20 (39%)	22 (43%)	
HP infection			0.7			0.6
Yes	14 (14%)	32 (16%)		8 (16%)	11 (22%)	
No	89 (86%)	171 (84%)		43 (84%)	40 (78%)	
Prior abdominal surgery			0.089			0.3
Yes	57 (55%)	90 (44%)		27 (53%)	21 (41%)	
No	46 (45%)	113 (56%)		24 (47%)	30 (59%)	
BPL (cm)	71.41 (22.58)	97.59 (14.81)	< 0.001	89.31 (17.55)	89.02 (12.37)	> 0.9
AL (cm)	141.41 (15.76)	141.87 (14.11)	0.8	136.57 (19.14)	137.75 (14.67)	0.8

Baseline demographics and clinical parameters for laparoscopic RYGB (L-RYGB) and robotic RYGB (R-RYGB) in the unmatched cohort and after 1:1 propensity score matching (PSM). *AL* Alimentary limb, *ASA* American Society of Anesthesiologists, *BMI* Body-mass index, *BPL* Biliopancreatic limb, *EBW* Excess body weight, *GERD* Gastroesophageal reflux disease, *OSAS* Obstructive sleep apnea syndrome, *HP* Helicobacter pylori

^1^ n (%); Mean (SD)

PSM resulted in 51 patients being matched for either the laparoscopic (PSM-L-RYGB) or robotic approach (PSM-R-RYGB). After PSM, baseline characteristics were comparable between groups. The proportion of male patients was similar (18% vs 20%; p > 0.9), and there was no significant difference in BPL length (89.31 ± 17.55 cm vs. 89.02 ± 12.37 cm; p = 0.8). Mean age at surgery did not differ between groups (45.10 ± 10.39 years vs. 44.73 ± 11.28 years; p = 0.9). Preoperative BMI was comparable (48.74 ± 7.08 kg/m^2^ vs. 48.59 ± 6.31 kg/m^2^; p > 0.9), as was excess body weight (EBW) (66.22 ± 21.50 kg vs. 65.70 ± 18.66 kg; p = 0.8).

The prevalence of comorbidities was likewise comparable in both matched groups (see Table 1), including rates of T2DM (35% vs. 39%; p = 0.8), arterial hypertension (49% vs. 61%; p = 0.3), steatosis hepatis (43% vs. 35%; p = 0.6), obstructive sleep apnea syndrome, gastroesophageal reflux disease, helicobacter pylori infection, hiatal hernia, and prior abdominal surgery. Overall, PSM resulted in sufficient comparability of the groups with respect to clinical baseline and procedural characteristics.

### Postoperative inflammation and MASLD trajectories

Leucocyte count and C-reactive protein (CRP) levels are summarized in Table 2. In both the unmatched and matched cohort, no significant differences in preoperative inflammatory status were observed between the groups. In the PSM cohort, preoperative leucocyte counts and CRP levels were comparable. Likewise, postoperative leucocyte counts and CRP-levels did not differ significantly between L- or R-RYGB. One year after surgery, both leucocyte counts and CRP levels were markedly reduced when compared to preoperative values, without significant intergroup differences.

**Table 2:** Inflammatory markers before and after propensity score matching (PSM)

Variable	Unmatched cohort			PSM cohort		
	L-RYGB N = 103^1^	R-RYGB N = 203^1^	p-value	L-RYGB N = 51^1^	R-RYGB N = 51^1^	p-value
**Leucocytes (giga/l)**						
Preoperative	8.43 (1.99)	8.20 (2.11)	0.2	8.30 (1.83)	8.21 (2.08)	0.8
Postoperative	9.94 (2.79)	9.79 (2.35)	0.8	10.21 (2.45)	9.47 (2.09)	0.2
3 M	7.20 (2.03)	7.14 (2.03)	0.6	7.15 (1.80)	7.17 (2.29)	> 0.9
6 M	7.45 (2.25)	7.15 (2.08)	0.2	7.28 (1.64)	7.10 (1.91)	0.5
1 a	6.99 (2.09)	6.70 (1.86)	0.3	6.89 (1.90)	6.55 (1.69)	0.2
**CRP (mg/l)**						
Preoperative	12.82 (10.73)	11.57 (9.73)	0.5	10.11 (6.49)	13.77 (14.34)	0.5
Postoperative	82.15 (57.93)	83.49 (53.93)	0.6	79.52 (55.75)	83.39 (44.94)	0.3
3 M	8.18 (10.91)	8.54 (10.49)	0.6	6.82 (10.21)	7.22 (6.46)	0.3
6 M	5.42 (6.79)	5.69 (10.91)	> 0.9	4.64 (5.55)	7.06 (19.41)	> 0.9
1 a	3.09 (7.20)	1.76 (2.69)	0.14	2.22 (3.66)	2.13 (2.76)	0.6

Leucocyte count and C-reactive protein (CRP) at preoperative baseline, postoperatively, and follow-up time points for L-RYGB and R-RYGB in unmatched and 1:1 PSM cohort. *CRP* C-reactive protein,* a* Years *M* Months

^1^ Mean (SD)

Trajectories of glycemic control and MASLD are depicted in Table 3. Both levels of HbA1c and NIT trajectories were comparable between groups in both the unmatched and matched cohorts at all timepoints. Both groups demonstrated a clinically meaningful reduction of HbA1c as early as 3 months, reflecting an improved glycemic control after MBS. NITs showed no significant differences between the PSM-L-RYGB and the PSM-R-RYGB group. Specifically, NAFLD fibrosis score (NFS) did neither differ preoperatively nor 1 year post-surgery, while demonstrating a reduction over time in all MBS patients, consistent with improvement of MASLD. 1 year after surgery, no significant differences were observed between PSM-L-RYGB and PSM-R-RYGB with respect to BMI, percentage of total weight loss, nor percentage of excess weight loss. Taken together, R- and L-RYGB showed comparable postoperative inflammatory and MASLD-related trajectories.

**Table 3:** Glycated hemoglobin (HbA1c), non-invasive tests for liver fibrosis (NITs), and weight loss before and after propensity score matching (PSM)

Variable	Unmatched cohort			PSM cohort		
	L-RYGB N = 103^1^	R-RYGB N = 203^1^	p-value	L-RYGB N = 51^1^	R-RYGB N = 51^1^	p-value
**HbA1c (%)**						
Preoperative	6.14 (1.00)	6.13 (1.03)	0.7	6.15 (1.10)	6.26 (1.10)	0.8
3 M	5.58 (0.68)	5.46 (0.65)	0.086	5.49 (0.64)	5.51 (0.61)	> 0.9
6 M	5.40 (0.62)	5.24 (0.56)	0.009	5.29 (0.49)	5.36 (0.61)	0.9
1 a	5.31 (0.65)	5.21 (0.46)	0.3	5.20 (0.56)	5.29 (0.44)	0.4
**APRI**						
Preoperative	0.23 (0.13)	0.26 (0.24)	0.5	0.25 (0.15)	0.24 (0.25)	0.4
3 M	0.30 (0.15)	0.31 (0.19)	> 0.9	0.33 (0.15)	0.28 (0.15)	0.3
6 M	0.26 (0.16)	0.27 (0.19)	0.4	0.29 (0.18)	0.28 (0.28)	0.5
1 a	0.26 (0.16)	0.27 (0.16)	0.2	0.28 (0.19)	0.26 (0.13)	> 0.9
**FIB-4**						
Preoperative	0.68 (0.35)	0.70 (0.42)	> 0.9	0.75 (0.37)	0.71 (0.44)	0.6
3 M	0.82 (0.38)	0.85 (0.45)	0.8	0.93 (0.41)	0.83 (0.43)	0.3
6 M	0.82 (0.42)	0.89 (0.89)	0.9	0.92 (0.43)	1.14 (1.62)	0.4
1 a	0.80 (0.40)	0.84 (0.44)	0.5	0.88 (0.46)	0.91 (0.51)	> 0.9
**NFS**						
Preoperative	-1.04 (1.38)	-1.08 (1.23)	0.8	-0.89 (1.46)	-1.08 (1.28)	0.5
3 M	-1.83 (1.30)	-1.73 (1.20)	0.5	-1.76 (1.29)	-1.79 (1.13)	0.9
6 M	-2.10 (1.28)	-1.84 (4.17)	> 0.9	-1.97 (1.30)	-0.99 (8.04)	0.8
1 a	-2.41 (1.20)	-2.41 (1.26)	> 0.9	-2.37 (1.32)	-2.25 (0.99)	0.6
**NI-NASH DS**						
Preoperative	1.85 (3.09)	2.37 (3.94)	0.6	2.06 (2.99)	2.05 (3.48)	0.8
3 M	-0.71 (3.81)	-1.23 (3.70)	0.2	-0.89 (3.23)	-1.13 (2.45)	> 0.9
6 M	-3.44 (4.30)	-3.53 (2.52)	0.4	-3.91 (5.17)	-3.55 (2.72)	0.8
1 a	-4.48 (4.13)	-5.12 (4.60)	0.2	-5.01 (4.77)	-5.01 (4.95)	0.8
**SAFE**						
Preoperative	3.45 (84.79)	4.15 (92.45)	> 0.9	16.80 (84.85)	-3.02 (97.46)	0.3
3 M	-3.08 (83.85)	2.52 (86.72)	0.6	10.79 (80.20)	-15.42 (80.39)	0.2
6 M	-34.91 (91.23)	-29.44 (89.64)	0.6	-20.48 (94.06)	-29.56 (102.11)	0.6
1 a	-65.04 (88.06)	-52.16 (96.81)	0.3	-50.08 (84.63)	-40.39 (79.30)	0.5
**BMI**						
3 M	39.78 (5.29)	39.36 (5.10)	0.5	39.09 (5.81)	39.37 (5.44)	0.8
6 M	35.01 (5.14)	34.94 (4.88)	> 0.9	34.38 (5.66)	35.26 (5.51)	0.5
1 a	31.55 (5.20)	31.02 (5.14)	0.2	30.97 (5.73)	31.78 (5.36)	0.4
**%TWL**						
3 M	18.56 (4.50)	19.00 (3.61)	0.5	19.25 (3.73)	18.96 (4.18)	0.8
6 M	28.27 (6.51)	28.15 (5.32)	0.9	28.95 (6.14)	27.71 (5.60)	0.2
1 a	35.66 (7.92)	36.06 (7.63)	0.7	36.49 (7.21)	34.76 (7.52)	0.14
**%EWL**						
3 M	39.33 (11.86)	40.51 (9.76)	0.3	41.69 (11.09)	40.57 (10.97)	0.8
6 M	59.90 (16.31)	59.81 (13.93)	0.8	62.78 (17.19)	59.30 (15.93)	0.3
1 a	75.06 (19.28)	76.64 (18.24)	0.2	78.65 (20.72)	74.07 (18.59)	0.2

*a Years, APRI* AST-to-Platelet Ratio Index, *BMI* Body-Mass-Index, *FIB-4* Fibrois-4 Index, HbA1c Glycated hemoglobin, *NFS* NAFLD-fibrosis score, *NI-NASH DS* Non-invasive NASH detection score, *SAFE* Steatosis-associated fibrosis estimator score, *%EWL* Percentage excess weight loss, *%TWL* Percentage total weight loss

^1^ Mean (SD)

### Intra- and postoperative outcome

Before PSM, intraoperative data revealed largely comparable findings between the L-RYGB and the R-RYGB group (see Table 4). However, hiatoplasty was performed less frequently in the L-RYGB than the R-RYGB group (2% vs 10%; p = 0.022). Length of surgery was significantly shorter in the L-RYGB group (98.03 ± 24.82 min vs. 121.07 ± 27.52 min; p < 0.001), whereas estimated blood loss was higher (66.65 ± 64.14 ml vs. 34.66 ± 43.45 ml; p < 0.001). After PSM, length of surgery remained the only intraoperative parameter that differed significantly between groups, being shorter in the PSM-L-RYGB group (100.94 ± 25.17 min vs. 131.33 ± 29.86 min; p < 0.001), while rates of adhesiolysis, hiatoplasty, and concomitant cholecystectomy were comparable.

**Table 4:** Intra- and postoperative characteristics before and after propensity score matching (PSM)

Variable	Unmatched cohort			PSM cohort		
	L-RYGB N = 103^1^	R-RYGB N = 203^1^	p-value	PSM-L-RYGB N = 51^1^	PSM-R-RYGB N = 51^1^	p-value
Adhesiolysis			0.7			> 0.9
Yes	7 (7.0%)	10 (5.0%)		4 (8.0%)	3 (6.0%)	
No	96 (93%)	193 (95%)		47 (92%)	48 (94%)	
Hiatoplasty			0.022			0.077
Yes	2 (2.0%)	20 (10%)		1 (2.0%)	7 (14%)	
No	101 (98%)	183 (90%)		50 (98%)	44 (86%)	
Cholecystectomy			0.4			0.2
Yes	4 (4.0%)	4 (2.0%)		0 (0%)	3 (6.0%)	
No	99 (96%)	199 (98%)		51 (100%)	48 (94%)	
Length of surgery (min)	98.03 (24.82)	121.07 (27.52)	< 0.001	100.94 (25.17)	131.33 (29.86)	< 0.001
Blood loss (ml)	66.65 (64.14)	34.66 (43.45)	< 0.001	50.49 (49.60)	38.24 (36.92)	0.2
Intraoperative complications			0.3			> 0.9
Yes	1 (1.0%)	0 (0%)		1 (2.0%)	0 (0%)	
No	102 (99%)	203 (100%)		50 (98%)	51 (100%)	
Pain while resting						
POD0	1.27 (1.26)	1.28 (1.64)	0.2	1.30 (1.53)	0.67 (0.71)	0.028
POD1	1.04 (1.02)	0.89 (1.22)	0.040	1.01 (1.18)	0.96 (1.03)	0.7
POD2	0.64 (0.83)	0.17 (0.52)	< 0.001	0.36 (0.61)	0.18 (0.44)	0.035
Pain in movement						
POD0	1.63 (1.18)	1.59 (1.83)	0.091	1.41 (1.25)	1.00 (1.02)	0.035
POD1	1.64 (1.37)	1.29 (1.38)	0.013	1.39 (1.45)	1.44 (1.07)	0.6
POD2	1.04 (0.95)	0.37 (0.64)	< 0.001	0.61 (0.75)	0.50 (0.69)	0.10
Non-opioid pain medication						
*POD0*			0.10			0.6
Yes	79 (78%)	136 (68%)		37 (76%)	41 (80%)	
No	22 (22%)	63 (32%)		12 (24%)	10 (20%)	
*POD1*			> 0.9			> 0.9
Yes	84 (82%)	165 (82%)		41 (80%)	40 (78%)	
No	19 (18%)	36 (18%)		10 (20%)	11 (22%)	
*POD2*			0.040			0.3
Yes	76 (74%)	123 (61%)		33 (65%)	27 (53%)	
No	27 (26%)	78 (39%)		18 (35%)	24 (47%)	
Opioids						
*POD0*			< 0.001			0.2
Yes	38 (38%)	23 (12%)		9 (18%)	4 (8.0%)	
No	63 (62%)	176 (88%)		40 (82%)	47 (92%)	
*POD1*			< 0.001			0.3
Yes	34 (33%)	19 (9.5%)		7 (14%)	3 (6.0%)	
No	69 (67%)	182 (91%)		44 (86%)	48 (94%)	
*POD2*			< 0.001			0.2
Yes	24 (23%)	5 (2.0%)		3 (5.9%)	0 (0%)	
No	79 (77%)	196 (98%)		48 (94%)	51 (100%)	
Postoperative complication			0.019			0.6
Yes	9 (9.0%)	5 (2.0%)		3 (6.0%)	1 (2.0%)	
No	94 (91%)	198 (98%)		48 (94%)	50 (98%)	
CD-Classification			0.027			0.4
0	96 (93%)	199 (98%)		49 (96%)	50 (98%)	
1	0 (0%)	2 (1.0%)		0 (0%)	1 (2.0%)	
2	3 (3.0%)	0 (0%)		1 (2.0%)	0 (0%)	
3a	1 (1.0%)	1 (0.5%)		0 (0%)	0 (0%)	
3b	2 (2.0%)	1 (0.5%)		1 (2.0%)	0 (0%)	
4a	1 (1.0%)	0 (0%)		0 (0%)	0 (0%)	
4b	0 (0%)	0 (0%)		0 (0%)	0 (0%)	
5	0 (0%)	0 (0%)		0 (0%)	0 (0%)	
Readmission to ICU			0.3			> 0.99
Yes	1 (1.0%)	0 (0%)		0 (0%)	0 (0%)	
No	102 (99%)	203 (100%)		51 (100%)	51 (100%)	
Length of stay (days)	4.48 (2.33)	3.20 (0.88)	< 0.001	3.75 (1.35)	3.20 (0.40)	0.002

Intraoperative and postoperative data for the L-RYGB and R-RYGB before and after PSM. *CD* Clavien- Dindo, *ICU* Intensive care unit, *POD* Postoperative day

^1^ n (%); Mean (SD)

Postoperative outcomes are summarized in Table 4. Before PSM, the L-RYGB group showed higher pain scores at rest on postoperative day (POD) 1 as well as during mobilization on POD1 and POD2. Administration of non-opioid pain medication was comparable between groups on POD0 and 1, but more frequent in the L-RYGB group on POD2. Opioid analgesics were administered more often in the L-RYGB early postoperatively. Postoperative complications occurred more frequently in the L-RYGB group (9% vs. 2%; p = 0.019) and required intervention more frequently. LOS was significantly longer in the L- than in the R-RYGB group (4.48 ± 2.33 days vs. 3.20 ± 0.88; p < 0.001). Before PSM, L-RYGB was associated with a unfavorable surgical outcome including higher pain levels and longer hospital stays due to more complications when compared to R-RYGB.

After PSM, most intra- and postoperative variables were comparable between both groups. However, pain while resting was persistently higher in the L- than in the R-RYGB group on POD0 (1.30 ± 1.53 vs. 0.67 ± 0.71; p = 0.028) and POD2 (0.36 ± 0.61 vs. 0.18 ± 0.44; < 0.035). Similarly, pain in movement was significantly higher in the L- than R-RYGB group on POD0 (1.41 ± 1.25 vs. 1.00 ± 1.02; p = 0.035). Moreover, LOS remained significantly higher in the L- compared to the R-RYGB group (3.75 ± 1.35 days vs. 3.20 ± 0.40 days; p = 0.002). Taken together, in the matched analysis, perioperative outcomes were largely comparable between both surgical approaches. Notably, selected parameters, including postoperative pain scores, the quantity and quality of pain medication, and LOS were beneficial after R-RYGB.

## Discussion

An ameliorated inflammatory response observed in minimally-invasive surgery, and particularly robotic surgery, translates into improved short- and long-term outcomes during oncological diseases such as gastric or colorectal cancer [26, 27]. However, whether a reduced postoperative inflammatory response associated with robotic surgery likewise improves MASLD in obese patients undergoing MBS remains uncertain. In this propensity-matched analysis, R-RYGB was neither associated with a reduced postoperative systemic inflammatory response nor with improved MASLD trajectories. Notably, R-RYGB was associated with lower postoperative pain and a shorter LOS.

As the unmatched cohort displayed imbalanced baseline characteristics, PSM was performed (see Fig. 2). Specifically, gender and BPL length were significantly different between both groups (see Table 1), both of which have been shown to affect short-term outcomes in MBS [28–30]. For instance, a longer BPL has been associated with improved %TWL and glycemic control five years after primary RYGB [29]. PSM reduces selection bias and the impact of confounders by balancing baseline covariates between groups, improving comparability and the validity of group comparisons [24, 25]. After PSM, baseline variables were comparable (see Table 1) displaying negligible absolute mean differences (see Fig. 2). Despite PSM, the retrospective design of this study remains vulnerable to unknown confounding.

The postoperative inflammatory response is influenced by patient-related and procedure-related factors, including surgical complexity and postoperative complications [31]. It is linked to short- and long-term outcome of patients undergoing MBS [12], with high CRP levels subsequent to MBS being associated with reduced weight loss and incomplete remission of T2DM [14, 32]. The relation of the postoperative inflammatory response after MBS and MASLD trajectories is unknown. We hypothesized that a robotic approach in patients receiving a RYGB attenuates the postoperative inflammatory response and thereby improves MASLD. This hypothesis was based on prior data from colorectal and gastric cancer surgery, in which robotic surgery has been associated with lower postoperative CRP levels compared with laparoscopy [7, 33, 34]. In these cases, an increased postoperative inflammatory response has been linked to unfavorable survival outcomes [27, 34, 35]. Contrary to our hypothesis, R-RYGB was not associated with a reduced postoperative inflammatory response. Since length of surgery correlates with postoperative CRP levels [36], one possible explanation for our observation could be that the length of surgery during L-/R-RYGB is markedly shorter when compared to complex colorectal (tumor) resections [37, 38]. Supporting this hypothesis, we likewise show that the length of surgery was significantly longer during R-RYGB compared to L-RYGB, which could potentially outbalance beneficial effects of R-RYGB on systemic inflammation. Taken together, our study does not support a clinically relevant effect of R-RYGB on the postoperative inflammatory response.

The absence of differences in postoperative inflammation was paralleled by comparable short-term outcomes between both surgical approaches, including MASLD trajectories. RYGB is an established and highly effective treatment for obesity and its metabolic comorbidities [39]. Although weight loss and treatment of T2DM appear comparable between L-RYGB and R-RYGB [40], data on the potential influence of R-RYGB on MASLD outcomes remain scarce. In our cohort, both approaches lead to substantial weight-loss, glycemic control and MASLD-related NIT trajectories at one year without differences between both approaches. This is consistent with previous reports on the therapeutic effect of MBS on obesity, T2DM, and MASLD [2, 39, 41]. The present study provides first insights into the impact of R-RYGB in comparison to L-RYGB on MASLD outcomes, which were unchanged. These findings are plausible, as postoperative inflammation, which is significantly elevated in MASLD and correlates with MASLD progression  [42, 43], was similar between both groups. Furthermore, the main drivers of partial or complete MASLD remission after MBS are weight-loss, improved insulin sensitivity, and changes in gut hormone and bile acid signaling, not the surgical-approach *per se* [44, 45]. Our findings should be interpreted in light of the use of NITs rather than histological liver assessment, which may not fully reflect the underlying state of hepatic histopathology.

Despite the comparable postoperative inflammatory response and short-term outcomes, perioperative outcome differed between both surgical approaches. Both laparoscopic and robotic surgery enhance postoperative recovery compared with open surgery. In RYGB, robotic surgery has been associated with a reduced LOS and improved postoperative pain [46], although, comparative propensity-matched studies remain limited. In this context, postoperative recovery as determined by pain scores and LOS were significantly improved in the R-RYGB group when compared to the L-RYGB group. These findings are consistent with previous work on the impact of robotic surgery in both MBS and other indications, suggesting that robotic surgery facilitate an improved recovery [47, 48]. The accelerated recovery observed in the R-RYGB group may have contributed to earlier discharge. Conclusively, these findings suggest that the main differences between R- and L-RYGB in our cohort relate to perioperative recovery rather than inflammatory or MASLD-related outcomes.

## Conclusion

Postoperative inflammation did not differ between L- and R-RYGB, suggesting no substantial effect of R-RYGB on the systemic inflammatory response. Short-term metabolic outcomes were comparable, with MASLD-related NITs displaying similar trajectories between both groups. R-RYBG was associated with extended length of surgery but a more favorable recovery profile during MBS. Thus, the metabolic benefits of RYGB are largely independent of the surgical approach, while R-RYGB may offer early recovery advantages.

## Data Availability

The data are available from the corresponding author upon reasonable request.
